# Resident Physician Intentions Regarding Unionization

**DOI:** 10.1001/jamanetworkopen.2025.3106

**Published:** 2025-04-03

**Authors:** Laura K. Barger, Matthew D. Weaver, Christopher P. Landrigan, Jason P. Sullivan, Rebecca Robbins, Ariel S. Winn, Charles A. Czeisler

**Affiliations:** 1 Division of Sleep and Circadian Disorders, Departments of Medicine and Neurology, Brigham and Women’s Hospital, Boston, Massachusetts; 2 Division of Sleep Medicine, Harvard Medical School, Boston, Massachusetts; 3 Division of General Pediatrics, Department of Pediatrics, Boston Children’s Hospital, Boston, Massachusetts; 4 Department of Pediatrics, Harvard Medical School, Boston, Massachusetts

## Abstract

**Importance:**

Resident physicians provide frontline care to approximately 70% of hospitalized patients. After decades of relative stability with a small minority of resident physicians unionized, unionization movements have succeeded in recent years at multiple major academic medical centers.

**Objective:**

To evaluate resident physicians’ unionization intention and the factors informing their unionization consideration at their institution.

**Design, Setting, and Participants:**

This nationwide survey study was conducted in May 2023 among a cohort of resident physicians, which was established over the course of 3 academic years (2020-2023). Resident physicians were invited to participate in an end-of-study survey on contemporary topics, which included questions on unionization.

**Exposure:**

Consideration of unionization by resident physicians.

**Main Outcomes and Measures:**

The main outcomes were the presence of a union at the institution for physicians in training, the presence of a movement to unionize, whether the resident physician would vote to unionize, and the factors most important in considering unionization. All measures were self-reported.

**Results:**

A total of 1235 participants (mean [SD] age, 28.8 [3.6] years; 737 of 1224 females [60%]; 791 of 1086 (73%) in their first postgraduate year) responded to survey questions regarding unionization. Twenty percent of participants (n = 249) reported that physicians in training belonged to a union at their institution. Among the 986 nonunionized resident physicians, 63% (625) reported they would vote to unionize, whereas less than 10% (96) would not vote to unionize. Pay and work hours were the most commonly cited factors in considering unionization (88% [1081 of 1235 participants] and 76% [941 of 1235 participants], respectively).

**Conclusions and Relevance:**

In this survey study, most resident physicians (>70%) reported either being in a union or supporting unionization at their institution, citing pay and financial security as critical factors in their consideration of unionization. Future research should investigate other factors and whether unionization achieves its goals of increased pay and benefits, work hours, and well-being.

## Key Points

**Question:**

What are resident physicians’ intentions toward unionization and what factors inform their consideration of unionization?

**Findings:**

In this nationwide survey study of 1235 resident physicians responding to questions about unionization, 20% reported working in unionized hospitals; among nonunionized resident physicians, 63% reported an intention to vote to unionize, and less than 10% reported an intention to vote against unionization. Pay and work hours were the most cited factors informing unionization decisions.

**Meaning:**

The findings indicate widespread support for unionization among resident physicians nationally who are not yet unionized and provide insight into the reasons underlying this rapidly growing movement.

## Teaser

This survey study examines the prevalence of, intentions toward, and factors considered in unionization among US resident physicians.

## Introduction

In the US, there are more than 150 000 resident physicians who provide much of the direct care in teaching hospitals.^1^ Resident physician compensation is far lower than that of senior physicians, particularly considering that resident physicians in most training programs routinely work many more hours per week compared with senior physicians. For the 3 to 7 years of their residency training, resident physicians are exempt from overtime compensation and have little to no opportunity to negotiate their work schedules, compensation, or benefits packages. Working conditions in this group, including hazardous extended-duration shifts, have been linked to adverse health, patient safety, and personal safety outcomes.^2,3,4^

Labor unions facilitate collective bargaining for employees to negotiate better working conditions. A large cross-sectional study of health care workers in varied job roles revealed union membership to be associated with substantially higher pay, better benefits, and a slightly greater number of total weekly work hours.^5^ The Committee of Interns and Residents (CIR), the first labor organization for resident physicians at public hospitals, was established in 1957 and merged with the much larger Service Employees International Union (SEIU) in 1997. Shortly thereafter, the National Labor Relations Board recognized resident physicians to have employee status at private hospitals as well, making it possible for all resident physicians to join labor unions.^6^ The prevalence of union membership among resident physicians remained relatively stable and low (≤10%) in the 1990s through 2010s, but it has been increasing over the past few years.^7,8^ This growing movement toward unionization among resident physicians was perhaps fueled by the work conditions during the COVID-19 pandemic.^9^ Among private employers, resident physicians voted for unionization in 4 institutions in 2022 and 7 institutions in 2023.^10^ For example, in 2022, 81% of resident physicians and fellows at Stanford voted in favor of unionization, with their first contract yielding a 21% increase in pay over 3 years.^11^ In 2023, 75% of resident physicians at Mass General Brigham voted in favor of joining CIR-SEIU, making it the largest resident physician union group in the US.^12^ In 2023, CIR-SEIU had approximately 31 000 members.^13^

This rapidly evolving unionization pattern remains poorly described at the national level, and factors informing the decisions of resident physicians to unionize have not been systematically assessed. As part of a larger nationwide study of resident physicians, an end-of-study survey was conducted that included questions regarding the state of unionization in this group. Specifically, we sought to evaluate resident physicians’ unionization intention and the factors informing their vote to unionize at their institution.

## Methods

A nationwide cohort study of US resident physicians was conducted from June 2020 to June 2023, with a primary objective of understanding the relationship between resident physician work hours, safety, and health. The research protocol was approved by the Mass General Brigham Institutional Review Board or historical equivalent (eg, Partners Healthcare). Each study participant provided electronic informed consent. We followed the American Association for Public Opinion Research (AAPOR) reporting guideline.

With the goal of reaching all resident physicians in the US, we used multiple strategies to recruit participants, including postings on social media; direct email invitations, if email addresses were publicly available; and forwarded invitations via medical school deans, program directors, and national resident organizations. Interested resident physicians were able to view study details and provide consent on the study website. Those who consented were emailed a baseline survey, which collected demographic information, including zip code and specialty (medical vs surgical). Thereafter, participants received monthly surveys, which collected information on work hours, health, and safety, for the duration of the academic years of the study. Race and ethnicity (self-reported as Asian, Black, Hispanic and non-Hispanic, White, multiracial, or other [including American Indian or Alaska Native, Native Hawaiian or Other Pacific Islander, and other]) were collected in the study to fulfill reporting requirements and to evaluate generalizability of the results.

Participants in the cohort study were invited to participate in the end-of-study survey, which was conducted in May 2023 to explore contemporary topics such as unionization of resident physicians. Questions included the following: Do physicians-in-training at your institution belong to a union?; Is there a movement to consider unionizing at your institution?; Would you vote to unionize, if a vote occurred?; and In thinking about unionization, what factors do you consider most important? An 8-point Likert scale was used to assess financial security, with the anchors being very financially insecure and very financially secure. To aid in the post hoc interpretation of results, we grouped financial security into quartiles.

### Statistical Analysis

Descriptive statistics were calculated for data on unionization and movement toward unionization at institutions as well as the intention to vote for unionization. Unpaired, 2-tailed *t* tests or χ^2^ analyses evaluated the differences between groups. Generalized linear models tested for differences in salary while controlling for potential confounders. Logistic regression ascertained whether demographics or current benefits were associated with a vote for unionization. Appropriate candidate factors were assessed based on relevance to the research question. Variance inflation factors were evaluated, and there was no evidence of collinearity. Responses that were missing key variables were omitted from those models (<1% missing sex, 12% missing postgraduate year, <1% missing specialty, and <5% missing region of the country). Patterns of missing data were assessed, and no imputation was performed.

All analyses were conducted using SAS, version 9.4 (SAS Institute Inc). Statistical significance was set at 2-sided *P* < .05.

## Results

A total of 5860 resident physicians consented to participate in the larger nationwide cohort study, of whom 4309 contributed at least 1 survey (74% cooperation rate) during 3 academic years (2020-2021, 2021-2022, and 2022-2023) and were invited to complete the end-of-study survey. Of 1334 residents who participated in the end-of-study survey (31% response rate), 796 of 1318 (60%) were female and 522 of 1318 (40%) were male, with a mean (SD) of 28.8 (3.8) years. Two hundred eighty-six of 1331 residents identified as Asian (21%), 60 of 1331 (5%) as Black, 88 of 1332 (7%) as Hispanic, 66 of 1331 (5%) as multiracial and 59 of 1331 (4%) identified as another race. Eight hundred forty-two of 1169 residents (72%) were in their first postgraduate year. Ultimately, 1235 participants (93%) responded to the questions about unionization, among whom 737 of 1224 were females (60%) and 487 were males (40%), with a mean (SD) age of 28.8 (3.6) years; 253 of 1235 identified as Asian (20%), 55 of 1235 as Black (4%), 84 of 1228 as Hispanic (7%), 813 of 1235 as White (66%), 58 of 1235 as multiracial (5%) individuals, and 58 of 1235 as having other (5%) race. Seven hundred ninety-one of 1086 (73%) were in their first postgraduate year. Table 1 shows the demographics of those who participated in the survey were mostly similar to those who did not participate and were comparable to the national population of resident physicians in Accreditation Council for Graduate Medical Education (ACGME)–accredited programs.^1^ The study cohort is slightly overrepresented in White resident physicians, those with medical specialties, and those identifying as female (Table 1).

**Table 1.** Resident Physician Demographics

Characteristic	Resident physicians in cohort, No./total No. (%)^a^		*P* value	No./total No. (%)^a^
	Participated in end-of-study survey	Did not participate in end-of-study survey		2022-2023 Resident physicians in ACGME-accredited programs
Sex				
Male	522/1318 (40)	866/2309 (38)	.21	82 164/158 079 (52)
Female	796/1318 (60)	1443/2309 (62)		75 480/158 079 (48)
Missing data	16	666		NA
Race^b^				
Asian	286/1331 (21)	573/2325 (25)	.03	46 767/158 079 (30)^c^
Black	60/1331 (5)	127/2325 (5)		11 433/158 079 (7)^c^
White	860/1331 (65)	1380/2325 (60)		85 515/158 079 (54)^c^
Multiracial	66/1331 (5)	118/2325 (5)		NR
Other^d^	59/1331 (4)	127/2325 (5)		7977/158 079 (5)^c^
Missing data	3	650		NA
Ethnicity^b^				
Hispanic	88/1322 (7)	224/2304 (10)	.002	9590/158 079 (6)
Non-Hispanic	1234/1322 (93)	2080/2304 (90)		NR
Missing data	12	671		NA
Specialty				
Medicine	1203/1324 (91)	2116/2313 (91)	.52	126 212/158 079 (80)
Surgical	121/1324 (9)	197/2313 (9)		30 374/158 079 (20)
Missing data	10	662		NA
Age at baseline, mean (SD), y	28.8 (3.8)	29.1 (4.5)	.04	30.9 (NR)^e^
Missing data	104	834		NA
PGY				
1	842/1169 (72)	1447/2018 (72)	.84	NA
≥2	327/1169 (28)	571/2018 (28)		NA
Missing data	165	957		NA

Abbreviations: ACGME, Accreditation Council for Graduate Medical Education; NA, not applicable; NR, not reported; PGY, postgraduate year.

^a^ Percentages were calculated without missing data.

^b^ Race and ethnicity were self-identified.

^c^ Four percent were unknown; multiple category was not provided.

^d^ Other included American Indian or Alaska Native, Native Hawaiian or Other Pacific Islander, and Other.

^e^ Including PGY 1 data only; no SD was provided.

Twenty percent of participants (249) reported that physicians in training belonged to a union at their institution. Those reporting being in a union had a significantly higher mean (SD) salary than those not belonging to a union ($70 271 [$7340] vs $65 455 [$8571]; *P* < .001), a difference that persisted after controlling for sex, postgraduate year, region of the country, and specialty. There was no difference in weekly mean (SD) work hours between unionized and nonunionized institutions (57.3 [11.8] vs 57.8 [0.8] hours).

**Figure 1.**
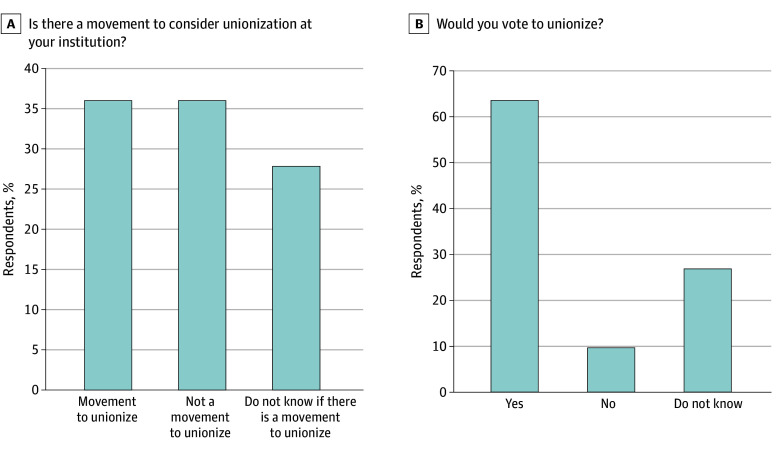
Percentage of Nonunionized Resident Physicians Reporting a Movement Toward Unionization at Their Institution and Whether They Would Vote for Unionization

Among the 986 nonunionized resident physicians, 36% (n = 360) reported that there was a movement to consider unionization at their institution. A similar percentage (36% [354]) reported that there was no movement to consider unionization at their institution, and 28% (276) reported that they did not know (Figure 1A). Sixty-three percent (625) reported they would vote to unionize, whereas less than 10% (96) would not vote to unionize and 27% (264) reported not knowing how they would vote (Figure 1B). Excluding undecided participants, females were more likely to vote for unionization than males (odds ratio [OR], 1.68; 95% CI, 1.08-2.62; *P* = .02). The odds of intention to vote for a union did not differ by race, ethnicity, or type of program (medical vs surgical).

Current unionization varied by US region. The West had the highest percentage of participants reporting unionized institutions at 42% (n = 106 of 252), followed by 25% (n = 86 of 348) in the Northeast, 9% (n = 26 of 301) in the South, and 8% (n = 22 of 276) in the Midwest (*P* < .001). Although 71% of participants in the West (n = 103 of 146) and 69% of participants in the Northeast (n = 181 of 264) reported they would vote for unionization, 61% of resident physicians in the Midwest (n = 154 of 253) and 58% in the South (n = 160 of 274) also indicated that they would vote for unionization (*P* = .03).

Lack of certain benefits was associated with increased odds of voting for unionization (Table 2). Resident physicians at institutions that did not provide childcare facilities or need-based stipends had an OR of 3.13 (95% CI, 1.38-7.09) or 3.41 (95% CI, 1.25-9.30), respectively, of voting for a union. Resident physicians had an OR of 2.10 (95% CI, 1.11-3.98) of voting for a union if their institution did not provide coverage when their children were sick.

**Table 2.** Association Between Lack of Specific Benefits and Likelihood of Voting for a Union

Benefits	OR (95% CI)	*P* value
Housing stipend	1.04 (0.61-1.77)	.88
Contribution to retirement fund	1.41 (0.91-2.18)	.12
Need-based stipends	3.41 (1.25-9.30)	.02
Paid maternity leave	1.69 (1.08-2.64)	.02
Paid paternity leave	1.77 (1.15-2.73)	.01
Childcare facilities	3.13 (1.38-7.09)	.01
Funding for childcare	1.80 (0.49-6.58)	.37
Coverage if your child is sick	2.10 (1.11-3.98)	.02
Flexibility to attend child’s doctors’ appointments	1.85 (1.10-3.11)	.02

Abbreviation: OR, odds ratio.

Increasing level of financial insecurity was associated with increased odds of voting for unionization. With the highest quartile of financial security on the Likert scale (very financially secure) as the referent, participants in the lowest quartile (very financially insecure) had an OR of 4.70 (95% CI, 1.88-11.77; *P* < .01) of voting for a union. Those in the third and second quartiles also had higher odds of intention to vote for a union (OR, 2.21 [95% CI, 1.22-4.01] and OR, 1.61 [95% CI, 0.94-2.78], respectively).

**Figure 2.**
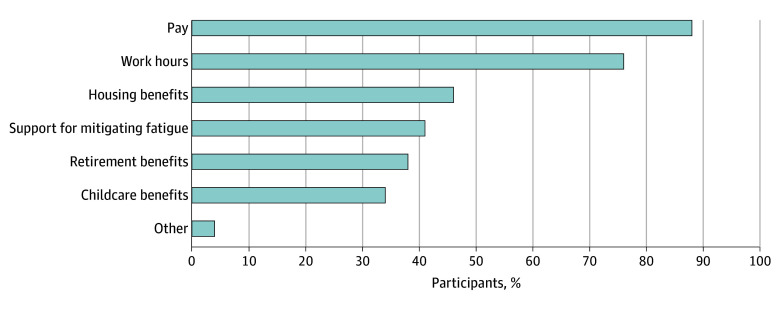
Factors Reported by Resident Physicians as Important in Thinking About Unionization

Pay was the most commonly cited factor in considering unionization (88% [1081 of 1235 participants]). Work hours were the second most frequently cited factor (76% [941 of 1235 participants]) (Figure 2). Although there was no difference by sex in the importance of pay in considering unionization, females more frequently than males cited work hours (81% [597 of 737] vs 69% [337 of 487]; *P* < .001) as an important factor; a larger proportion of females also cited childcare benefits, although the difference was not statistically significant (37% [269 of 737] vs 31% [154 of 487]; *P* = .08). Support for mitigating fatigue (eg, nap rooms, ride share) was also viewed differently between sexes, with 45% of females (329 of 737) and 35% of males (172 of 487) rating this factor as important in considering unionization (*P* < .001).

## Discussion

In this nationwide survey of resident physicians, 20% of participants reported that their institution was unionized, and 29% had a movement considering unionization. Among nonunionized resident physicians, 63% indicated that they would vote for unionization and less than 10% indicated that they would vote against it. To our knowledge, these findings from a nationally representative cohort of resident physicians are the first to be published regarding intentions to unionize and the factors informing these decisions. These data are remarkably similar to a recent report by Ahmed and Li^10^ cataloging actual National Labor Relations Board elections at academic medical centers, in which only 16.5% of eligible resident physicians voted against unionization. Our finding that 20% of resident physicians across the US are unionized, approximately double past unionization rates, is consistent with the 2023 membership report of the CIR-SEIU.^13^

Most participants cited pay (currently the equivalent of approximately $20 to $25 per hour) as the most important factor in considering a vote for unionization, and the least financially secure resident physicians were more than 4 times as likely to vote for unionization. Similarly, resident physicians were 3 times as likely to favor unionization if their institution did not provide need-based stipends. Need-based stipends for economically disadvantaged resident physicians have become increasingly common, and their provision aims to address a key social barrier to medical education.^14^ Increased salary and housing stipends were the most commonly cited union benefits in interviews with surgical residents.^15^ This response may stem from recent increases in cost of living nationwide, including a 33% increase in housing prices over the past 5 years.^16,17^ The geographical distribution of resident physician unionization and intent to vote for unionization were similar to that of all union members, with the majority working in the West and Northeast.^18^ It is unclear if unionization in those regions is associated with the higher cost of living or political factors, such as right-to-work legislation.

Resident physicians at unionized institutions had a 7% higher salary than those at nonunionized institutions. Higher salaries were also reported by the Bureau of Labor Statistics for all types of unionized workers.^18^ In a study of all health care workers, overall those in unions had higher salaries, although there was no difference between unionized and nonunionized physicians and dentists (grouped together).^5^ Additionally, health care workers at unionized institutions have more benefits, including pensions.^5^ Pay and financial security may become even greater considerations as institutions strive to build a more diverse physician workforce, as those from groups underrepresented in medicine often acquire more debt during their medical training.^19^

Work hours (currently averaging nearly 60 hours per week) were the second most frequently cited factor important to participants’ consideration of unionization. Tolerance for working long hours may be lower than in previous years due to increasing discussion about work-life balance, disillusionment with the medical system, and a postpandemic world in which many of their peers in other professions have more flexible work schedules or arrangements, such as work from home.^13,20^ There was no difference in work hours between resident physicians at unionized and nonunionized institutions. In 1 study of all health care workers, those at unionized institutions had longer work hours, on average.^5^ Data from Ahmed and colleagues^5^ reveal that among resident physicians who voted on unionization between 2013 and 2017, when the ACGME imposed a 16-hour work-hour limit for first-year residents, 51.5% voted against unionization. In contrast, among resident physicians who voted on unionization after the ACGME work-hour restriction was rescinded, only a minority (20.6%) of resident physicians cast their vote against unionization.^5^ In the present survey, the less than 10% of participants who indicated they would vote against unionization substantiates a growing pattern that has not been quantified and suggests that changes in work-hour policy limits may have played a role in the perceived need for resident physician unions.

Although workload was not included in the list of factors important in considering unionization, it should be acknowledged as a potential contributing factor.^21^ The additional work associated with electronic medical records, patient portals, and the corporatization of health systems, such as an emphasis on work productivity, has been cited as a factor in physician burnout, and these factors’ association with unionization requires further investigation.^22,23^

Resident physicians working in institutions without paid parental leave and without childcare benefits are 3 times more likely to vote for unionization. Resident physicians have become slightly older in recent years, and there is more female representation, likely resulting in a workforce with existing family responsibilities.^13^ Perhaps unsurprisingly, given that historically housework and childcare responsibilities were fulfilled by mothers,^24^ women more frequently cited work hours and childcare as important factors in their unionization vote compared with men. Although historically and in the 2023 Bureau of Labor Statistics report, men have a higher level of union membership,^18^ we found that women are more likely than men to vote for unionization. Prior research has also shown that women were more likely to vote for unionization than men, seeking higher wages and more participation in decision-making.^25,26^

Critics claim that physicians who unionize are putting economic advantage first and may compromise physician-patient relationships.^14^ Surgical resident physicians and their leadership suggest that resident physicians may not be able to take advantage of all union benefits due to staffing issues and that unionization may interfere with the primary mission of residency as a training program.^15^ The US Supreme Court, however, in a unanimous decision written by Chief Justice John Roberts, solidified resident physicians’ primary status as employees in 2011.^27^

### Limitations

This study has some limitations. Although the response rate for the end-of-study survey was 31%, the overall cooperation rate was 74%. Participants were largely representative of US resident physicians; the demographics of those who completed the end-of-study survey are comparable to nonparticipants in the full cohort and to all resident physicians nationwide. The risk of nonresponse bias was low given that the unionization questions were embedded within the end-of-study survey, with a focus different from the main study’s topics. However, participants may reflect those with a particular interest in the main study’s focus of work hours, health, and safety. The survey questions regarding unionization intention and voting factors were not validated in prior studies nor formally tested for reliability. Face validity was assessed through an iterative process with leaders of a residency program at a Harvard Medical School–affiliated hospital. Participants were not required to answer every question. The comparison of the association of benefits with likelihood of voting for a union may be limited due to the rarity of some of these benefits (eg, need-based stipends) and the majority of participants who favored voting for a union. As a result, there were small cell sizes for some comparisons and subsequent unstable estimates of the precise association between availability of specific benefits and likelihood of voting for a union. Additionally, this study relied on self-reports of work hours and salary, which potentially introduces recall bias. Future studies should consider objective measures, such as time sheets or pay stubs.

## Conclusions

The results of this study substantiate the increasing prevalence of unionization among resident physicians, who traditionally work long hours with low hourly compensation. For resident physicians answering the unionization questions in the end-of-study survey, pay and work hours were the 2 most important factors in their unionization decisions. Future research should investigate other factors, such as workload, electronic health records, and patient portals, in unionization considerations; whether unionization leads to increased financial stability, reduced work hours, and provision of other benefits; and whether such changes are associated with improved work-life balance and decreased rates of burnout.

## Notes

Supplement.
Data Sharing Statement
