# Supplementary material for: Resident Physician Intentions Regarding Unionization
Source: JAMA Netw Open. 2025 Apr 3;8(4):e253106. doi: 10.1001/jamanetworkopen.2025.3106 (PMC11969282; doi:10.1001/jamanetworkopen.2025.3106)
Supplement: Supplement. — Data Sharing Statement [file jamanetwopen-e253106-s001.pdf]

Data Sharing Statement

Barger. Resident Physician Intentions Regarding Unionization. *JAMA Netw Open*. Published April 03, 2025. doi:10.1001/jamanetworkopen.2025.3106

Data
Data available: Yes
Data types: Deidentified participant data
How to access data: Data will be shared upon reasonable request and in accordance with IRB policy. Contact corresponding author.
When available: beginning date: 01-01-2026

Supporting Documents
Document types: None

Additional Information
Who can access the data: Data will be shared upon reasonable request and in accordance with IRB policy.
Types of analyses: Data will be shared upon reasonable request for secondary analysis and in accordance with IRB policy.
Mechanisms of data availability: Mechanism will include a signed data agreement.
